# C-Cbl reverses HER2-mediated tamoxifen resistance in human breast cancer cells

**DOI:** 10.1186/s12885-018-4387-5

**Published:** 2018-05-02

**Authors:** Wei Li, Ling Xu, Xiaofang Che, Haizhou Li, Ye Zhang, Na Song, Ti Wen, Kezuo Hou, Yi Yang, Lu Zhou, Xing Xin, Lu Xu, Xue Zeng, Sha Shi, Yunpeng Liu, Xiujuan Qu, Yuee Teng

**Affiliations:** 1 Department of Medical Oncology, the First Hospital of China Medical University, Shenyang, 110001 Liaoning China; 2 Key Laboratory of Anticancer Drugs and Biotherapy of Liaoning Province, the First Hospital of China Medical University, NO. 155, North Nanjing Street, Heping District, Shenyang, 110001 Liaoning China; 3 Jinzhou Center Hospital, Jinzhou, 121000 Liaoning China; 4 Laboratory Animal Center, China Medical University, Shenyang, 110001 Liaoning China

**Keywords:** Breast cancer, Tamoxifen, Resistance, HER2, C-Cbl

## Abstract

**Background:**

Tamoxifen is a frontline therapy for estrogen receptor (ER)-positive breast cancer in premenopausal women. However, many patients develop resistance to tamoxifen, and the mechanism underlying tamoxifen resistance is not well understood. Here we examined whether ER-c-Src-HER2 complex formation is involved in tamoxifen resistance.

**Methods:**

MTT and colony formation assays were used to measure cell viability and proliferation. Western blot was used to detect protein expression and protein complex formations were detected by immunoprecipitation and immunofluorescence. SiRNA was used to examine the function of HER2 in of BT474 cells. An in vivo xenograft animal model was established to examine the role of c-Cbl in tumor growth.

**Results:**

MTT and colony formation assay showed that BT474 cells are resistant to tamoxifen and T47D cells are sensitive to tamoxifen. Immunoprecipitation experiments revealed ER-c-Src-HER2 complex formation in BT474 cells but not in T47D cells. However, ER-c-Src-HER2 complex formation was detected after overexpressing HER2 in T47D cells and these cells were more resistant to tamoxifen. HER2 knockdown by siRNA in BT474 cells reduced ER-c-Src-HER2 complex formation and reversed tamoxifen resistance. ER-c-Src-HER2 complex formation was also disrupted and tamoxifen resistance was reversed in BT474 cells by the c-Src inhibitor PP2 and HER2 antibody trastuzumab. Nystatin, a lipid raft inhibitor, reduced ER-c-Src-HER2 complex formation and partially reversed tamoxifen resistance. ER-c-Src-HER2 complex formation was disrupted by overexpression of c-Cbl but not by the c-Cbl ubiquitin ligase mutant. In addition, c-Cbl could reverse tamoxifen resistance in BT474 cells, but the ubiquitin ligase mutant had no effect. The effect of c-Cbl was validated in BT474 tumor-bearing nude mice in vivo. Immunofluorescence also revealed ER-c-Src-HER2 complex formation was reduced in tumor tissues of nude mice with c-Cbl overexpression.

**Conclusions:**

Our results suggested that c-Cbl can reverse tamoxifen resistance in HER2-overexpressing breast cancer cells by inhibiting the formation of the ER-c-Src-HER2 complex.

**Electronic supplementary material:**

The online version of this article (10.1186/s12885-018-4387-5) contains supplementary material, which is available to authorized users.

## Background

Breast cancer is one of the most common malignant tumors in women and the second major cause of cancer-related death in women [1]. The estrogen receptor (ER) is a member of the nuclear hormone receptor family, which plays an important role in cell proliferation, differentiation, and tumor formation. Approximately 60%–70% of breast tumors are ER-positive at diagnosis, and anti-estrogen therapies, such as tamoxifen, are very important in premenopausal women breast cancer management [2]. However, nearly 50% of breast cancer patients develop resistance to endocrine therapy, leading to tumor progression and reduced patient survival [2]. Therefore, research on the mechanism of tamoxifen resistance is important to improve the prognosis of breast cancer patients.

Human epidermal growth factor receptor 2 (HER2)/neu overexpression or amplification is found in approximately 15%–30% of breast cancers, and increased expression of this receptor correlates with poor clinical outcome and resistance to endocrine therapy [2, 3]. The crosstalk between ER and HER2 pathways plays an important role in both intrinsic and acquired resistance to endocrine therapy [4], and thus current research in drug resistance has mainly focused on these pathways.

Evidence indicates that the non-genomic actions of estrogen are mediated by membrane-associated ERα, which resides in or near the cell membrane and interacts with several growth factor signaling pathways [5]. A previous study showed that extranuclear ER colocalizes with the HER2 receptor in membrane signaling domains that modulate downstream nuclear events leading to the growth of breast cancer cells [3].

Lipid rafts provide a functional platform for the interaction of protein molecules through dynamic aggregation [6]. Lipid rafts are essential for the plasma membrane localization of ER and play a critical role in its membrane-initiated effects [7, 8]. In HER2-overexpressing breast cancer cells, HER2 receptors are present on the cell surface as monomers, homodimers, and heterodimers [9]. Signal activation and transduction require the localization of HER2 to lipid rafts, and lipid raft aggregation is necessary for HER2 activation and function. Combination of lipid rafts inhibition, tamoxifen was more effective in inhibiting the proliferation of melanoma cells [10]. Cholesterol-rich lipid rafts were highly amplified in TAM resistant cell lines, disrupted lipid rafts acted cooperatively with TAM to reduce prosurvival mediators [11]. However, whether HER2 interacts with ER in lipid rafts and whether this interaction is involved in tamoxifen resistance remains unclear.

Research has shown that the molecular mechanisms of estrogen involve the ability of the 17beta-estradiol (E2)-ER complex to induce gene transcription through specific coregulators (i.e. coactivators or corepressors) [12, 13] and to evoke membrane-initiated activation of specific rapid phosphorylation cascades, such as Src/ERK/MAPK [13, 14]. A previous study showed that E2-induced Src activation requires the formation of a protein complex that contains at least ERα and c-Src [15]. In previous reports, membrane-associated ERα was shown to interact with HER2 in the presence of E2 [16], and HER2 interacts with the non-receptor tyrosine kinase c-Src [17]. No studies have examined the complex formation between HER2, ERα and c-Src.

Previous studies show that the ubiquitin ligases c-Cbl and Cbl-b are important regulators of lipid rafts [18]. Cbl-b inhibits the aggregation of T cell receptors by preventing lipid raft aggregation, and we previously showed that Cbl-b inhibits aggregation of lipid rafts [18]. Recent work from our group also showed that c-Cbl and Cbl-b overexpression inhibits lipid raft aggregation in gastric cancer cells [19]*.*

In this study, we hypothesized that the ER-c-Src-HER2 complex formation is involved in HER2-mediated tamoxifen resistance and that lipid rafts are important sites for the formation of these complexes. We also investigated the potential role of c-Cbl in modulating the ER-c-Src-HER2 complex formation and function through its established effects on lipid raft formation.

## Methods

### Reagents and antibodies

Anti-HER2 was purchased from NeoMarker (Waltham, MA, USA), anti-ER, anti-PR, anti-c-Src, anti-p-HER2 (Tyr1248), anti-p-c-Src (Tyr416), and anti-p-ER (Ser118) were purchased from CST (Danvers, MA, USA). Anti-actin, anti-c-Cbl, anti-Caveolin1 and anti-HER2 labeled with phycoerythrin were obtained from Santa Cruz Biotechnology (Dallas, TX, USA). Tamoxifen, 17-β estradiol, Nystatin, PP2were purchased from Sigma (St. Louis, MO, USA). The c-Cbl-3 × flag-CMV9 plasmid was constructed by Taihe Biotechnology (Beijing, China). The HER2-pEGFP-N1 plasmid was constructed by GENEWIZ (Suzhou, China). The PSVL-cbl-70Z plasmid was kindly gift from Professor Kiyonao Sada (Kobe University Graduate School of Medicine). The lentiviral system was purchased from Genechem (Shanghai, China).

### Cells and cell culture

The breast cancer BT474 and T47D cell lines were obtained from Cell Bank of Type Culture Collection of Chinese Academy of Sciences (Shanghai, China). BT474 (catalogue number TCHu143) is an ER-positive and HER2-overexpressing cell line. BT474 cells were cultured in RPMI 1640 medium (Hyclone, GE, USA) containing 10% fetal bovine serum (FBS), penicillin (100 U/mL), and streptomycin (0.1 mg/mL). T47D (catalogue number TCHu87) is an ER-positive and low HER2-expressing cell line. T47D cells were maintained in DMEM (Hyclon, GE, USA) containing 10% fetal bovine serum (FBS), penicillin (100 U/mL), and streptomycin (0.1 mg/mL). Cells were cultured at 37 °C in 5% CO_2_. The cells were routinely subcultured every 3–5 days, and cells used for experiments were from the logarithmic growth phase. All cell lines were mycoplasma negative by PCR reaction.

### Cell viability assay

Cell proliferation was measured with the 3-(4, 5-dimethyl thiazol-2-yl)-2, 5-diphenyl tetrazolium bromide (MTT) assay. Cells (5 × 10^3^ cells/well) were seeded in 96-well plates for 24 h and then treated with E2 (10 nmol/L) and/or tamoxifen (1 μmol/L). Absorbance was measured at 570 nM at 48 and 72 h. The data represents the mean ± SD of at least nine wells from three independent experiments.

### Colony formation analysis

Cells were plated in 6-well plates (800 cells/well), and after 24 h the cells were treated with E2 (10 nmol/L) and/or tamoxifen (1 μmol/L). Treated cells were then cultured for another 14 days. After removing the medium from the wells, colonies were stained with Giemsa and images were captured.

### Western blot analysis

Western blot analysis was performed as described in our previous studies [20]. Cells were solubilized in 1% Triton lysis buffer and quantified with the Kaumas blue method. Cell lysates were separated by SDS-PAGE and transferred to a PVDF membrane. The PVDF membranes were blocked by 5% skim milk powder in TBST buffer for 2 h at room temperature. The membranes were then incubated with primary antibodies overnight (HER2 1:1000, p-HER 1:500, ER 1:1000, p-ER 1:500, c-Src 1:1000, p-c-Src 1:250, c-Cbl 1:1000, Caveolin1 1:250, Actin 1:1000) at 4 °C, followed by incubation with secondary antibodies(CST, 1:2000) for 30 min at room temperature. The proteins were detected with enhanced chemiluminescence reagent (SuperSignal Western Pico Chemiluminescent Substrate; Pierce, USA) and visualized with the Electrophoresis Gel Imaging Analysis System (Hercules, California, USA).

### Immunoprecipitation

Immunoprecipitates was performed as described in our previous studies [21]. Briefly, the collected cell lysates were incubated with anti-c-Src antibody or immunoglobulin-G (CST), and precleared protein G-agarose beads for 6 h at room temperature. Immunoprecipitates were washed four times in mild lysis buffer and then subjected to western blot analysis using anti-HER2(1:1000), anti-ER(1:1000), and anti-c-Src antibodies(1:1000).

### Inhibition of lipid rafts

Lipid rafts are dynamic assemblies of proteins and lipids that harbour many receptors and regulatory molecules and so act as a platform for signal transduction, nystatin inhibits lipid rafts by affecting lipid metabolism [22]. To inhibit the function of lipid rafts, cells were treated with nystatin (5 μg/mL) for 2 h before the experiments.

### HER2 siRNA

Three siRNAs against HER2 and a scrambled control were purchased from VIEWSOLID BIOTECH (Beijing, China) The si-HER2 target sequences were as follows:5′-GUUGGAUGAUUGACUCUGATT-3′,5′-GGAGACCCGCUGAACAAUATT-3′, and 5′-GCUCAUC GCUCACAACCAATT-3′. SiRNA transfection was performed using Lipofectamin 2000 (Invitrogen, Carlsbad, CA, USA), according to the protocol previously [21].

### Cell transfection and lentiviral infection

Plasmid transfection was performed using Lipofectamine 2000, according to the protocol previously [21]. For in vivo experiments, c-Cbl was overexpressed with a lentiviral system. Lentiviral production and infection were performed following the standard procedure recommended by the company (Shanghai Genechem Co., Ltd.). At 72 h, the virus-infected cells were used for experimental analysis.

### In vivo xenograft animal model

Pathogen-free female BALB/c nude mice (4 weeks of age) were purchased from WeiTongLiHua (Beijing, China). The animals were housed in accordance with institutional ethical guidelines of animal care. Mice were housed in specific pathogen-free conditions, three per cage, and maintained at constant temperature (22 °C) and humidity. At 5 weeks of age, the mice were randomized into two groups of six mice each. To establish xenograft tumors, a breast cancer cell suspension (1 × 10^7^ cells in 0.2 mL of PBS was injected subcutaneously in the right flank of each nude mouse. One group was subcutaneously inoculated with BT474 cells infected with lentiviral vector control and the second group was inoculated with BT474 cells infected with c-Cbl lentivirus. Tumor growth was measured with fine calipers twice each week and the tumor volume was calculated by the formula shown below:

$$ V=\left(L\times {W}^2\right)/ 2 $$

We observed 100% tumor incidence for both groups. When the volume of xenografts reached 50–100 mm^3^, each group was divided into two treatment subgroups: vehicle control or tamoxifen treatment (three mice per group). The control groups received 200 μL saline daily from days 1–7. The tamoxifen gavage group received tamoxifen (20 mg/kg) daily from days 1–7. Tumor volume and body weight were measured twice per week. Treatments continued for 7 days, and then mice were observed for 1 week. At the end of the experiment, all mice were humanely euthanized and necropsied. Tumor tissues were harvested, rinsed in saline, weighed, and immediately formalin-fixed.

### Immunofluorescence

Tumor samples were formalin-fixed, paraffin-embedded and then cut into 4 μm sections. All sections were de-paraffinized in xylene and hydrated through a graduated alcohol series and then immunofluorescence was performed based on standard procedures [21]. The sections were permeabilized with 0.2% Triton X-100 for 5 min, blocked with 5% bovine serum albumin for 1 h and then incubated with phycoerythrin-labeled anti-HER2 (Sc-33684PE), anti-c-Src (SC8056), and anti-ER (SC543) antibodies (all purchased from Santa Cruz) overnight at 4 °C. The next day, Alexa Fluor 405-conjugated goat anti-mouse IgG or Alexa Fluor 488-conjugated goat anti-rabbit IgG (Molecular Probes) antibodies were added in blocking solution for 1 h at room temperature in the dark. DAPI (4′6′-diamidino-2 phenylindole) was used to stain nuclei for 1 min. After mounting with the Slow Fade Antifade Kit (Molecular Probes, Eugene, OR, USA), the sections were visualized by fluorescence microscopy (BX61, Olympus, Japan).

### Statistical analysis

All experiments were performed at least in triplicate and repeated in three independent studies. The data are expressed as the mean ± SD. Differences between groups were compared using the Student’s t-test. SPSS Statistics 17.0.1 (SPSS, Chicago, IL, USA) was used for statistical analysis. *P* < 0.05 was considered significant.

## Results

### HER2-overexpressing breast cancer cells are resistant to tamoxifen

**Fig. 1:**
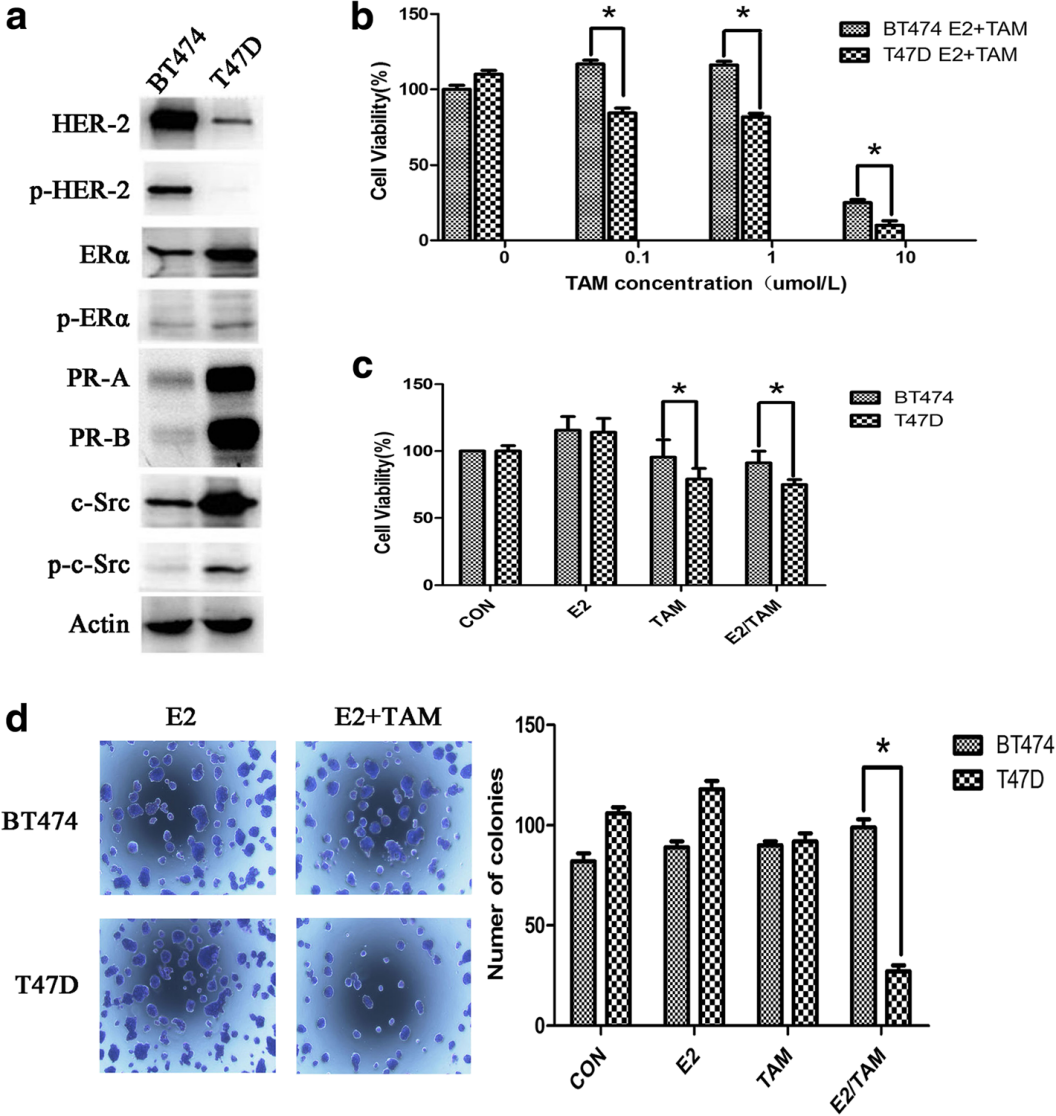
BT474 cells are resistant to tamoxifen. **a** Characterization of BT474 and T47D cells. Western blot analysis of ER, HER2, PR, and c-Src and their phosphorylation status in both cell lines, and Actin is as a loading control. **b** BT474 and T47D cells were seeded in 96-well plates at a density of 5000 cells/well and then treated with 4-OH tamoxifen (0–10 μmol/L). After 3 days, MTT assays were performed. **c** BT474 and T47D cells were seeded in 96-well plates and 24 h after plating, cells were treated with vehicle (CON), 17β-E2 (E2) (10 nmol/L), tamoxifen (TAM) (1 μmol/L), or combination (E2/TAM). After 48 h, MTT assays were performed. The bar graph shows the sensitivity of cells to the indicated treatments. Data represent the mean ± SD of at least three independent experiments. **d** Effect of tamoxifen on colony formation in BT474 cells. After 14 days, colonies in 6-well plates were imaged and quantified by counting the number of colonies. At least nine wells per treatment group were quantified per value reported. The results show the mean from three independent experiments. *P < 0.05

We first performed molecular characterization of the BT474 and T47D breast cancer cell lines, as shown in Fig. 1a. BT474 cells showed positive ER expression and high HER2 expression, whereas T47D cells showed positive ER expression and low HER2 expression. We next examined tamoxifen sensitivity of the two cell lines. Cell viability assays and colony formation assays demonstrated that BT474 cells were more resistant to tamoxifen than T47D cells (Fig. 1b–d), indicating that breast cancer cells with HER2 overexpression were resistant to tamoxifen.

### Detection of the ER-c-Src-HER2 complex in tamoxifen-resistant BT474 cells

**Fig. 2:**
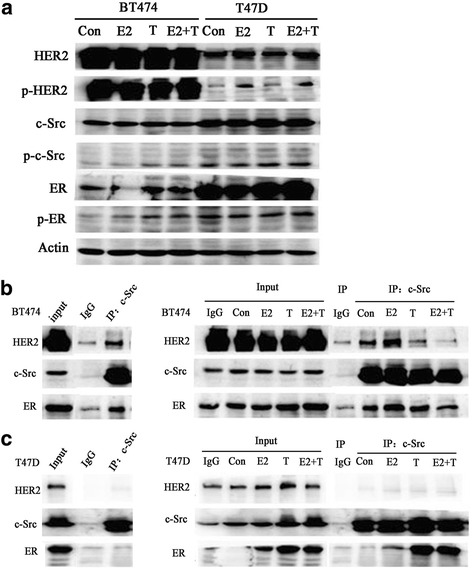
ER-c-Src-HER2 complex formation in the tamoxifen-resistant breast cancer cell line BT474. **a** BT474 or T47D cells (approximately 60% confluent) were treated with 17β-E2 (10 nmol/L) and/or TAM (1 μmol/L) or left untreated (vehicle control) for 4 h. Lysates were prepared and subjected to immunoblot analysis with the indicated antibodies. **b** BT474 and (**c**) T47D cells were treated with 17β-E2 (10 nmol/L) and/or tamoxifen (TAM) (1 μmol/L) for 4 h, and then lysates were immunoprecipitated using a c-Src antibody or IgG and subjected to immunoblot analysis using the indicated antibodies

Western blot analysis showed that the level of phosphorylated HER2 (p-HER2) is higher in BT474 cells compared with T47D cells (Fig. 2a). Treatment of E2 cells also resulted in activation of HER2 in T47D. However, the activation of HER2 in T47D cells did not result in resistance to tamoxifen, and this may be due to the low expression of HER2 in these cells.

We next evaluated whether ER, c-Src and HER2 formed a complex in breast cancer cells. Immunoprecipitation analysis confirmed the ER-c-Src-HER2 complex in tamoxifen-resistant BT474 cells, whereas the complex was not detected in tamoxifen-sensitive T47D cells (Fig. 2b and c). To determine the impact of estrogen or tamoxifen on complex formation, cells were treated with estrogen or tamoxifen alone or in combination for 4 h and then evaluated for the formation of the ER-c-Src-HER2 complex. Estrogen, tamoxifen and the combination treatment all promoted the formation of the ER-c-Src-HER2 complex in BT474 cells. In T47D cells, estrogen and/or tamoxifen treatment did not promote the formation of the ER-c-Src-HER2 complex. However, tamoxifen did induce binding between ER and c-Src. These results indicated that the level of p-HER2 is elevated in BT474 cells and that HER2, c-Src, and ER can form a complex in BT474 cells.

### Overexpression of HER2 leads to ER-c-Src-HER2 formation in T47D cells

**Fig. 3:**
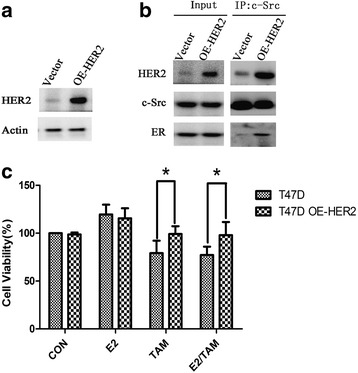
Overexpression of HER2 leads to tamoxifen resistance in T47D cells. **a** T47D cells were transfected with HER2-pEGFP-N1 plasmid (OE-HER2) or pEGFP-N1 vector as a control (Vector). Immunoblot analysis was performed using the indicated antibodies. **b** T47D cells were transfected with the HER2 overexpression vector for 48 h, and immunoprecipitation and immunoblot analysis were performed with the indicated antibodies. **c** MTT assays in HER2-overexpressing T47D cells treated with 17β-E2 and/or tamoxifen. T47D cells were transfected with pEGFP-N1 vector or HER2-pEGFP-N1 (OE-HER2) for 24 h, and then treated with vehicle (CON), 17β-E2 (E2) (10 nmol/L), tamoxifen (TAM) (1 μmol/L) or combination treatment (E2/TAM) for another 48 h. MTT assays were then performed. *P < 0.05

We next examined the role of HER2 in impacting ER-c-Src-HER2 complex formation and function. We transfected T47D cells with a HER2 expression vector and confirmed elevated HER2 levels (Fig. 3a). Overexpression of HER2 in T47D cells led to ER-c-Src-HER2 complex formation (Fig. 3b). Furthermore, overexpression of HER2 resulted in the T47D cells becoming more resistant to tamoxifen (Fig. 3c). These results indicated that the level of HER2 in T47D cells is related to tamoxifen resistance.

### Inhibition of HER2, c-Src reduced ER-c-Src-HER2 complex formation and reversed the resistance of BT474 cells to tamoxifen

**Fig. 4:**
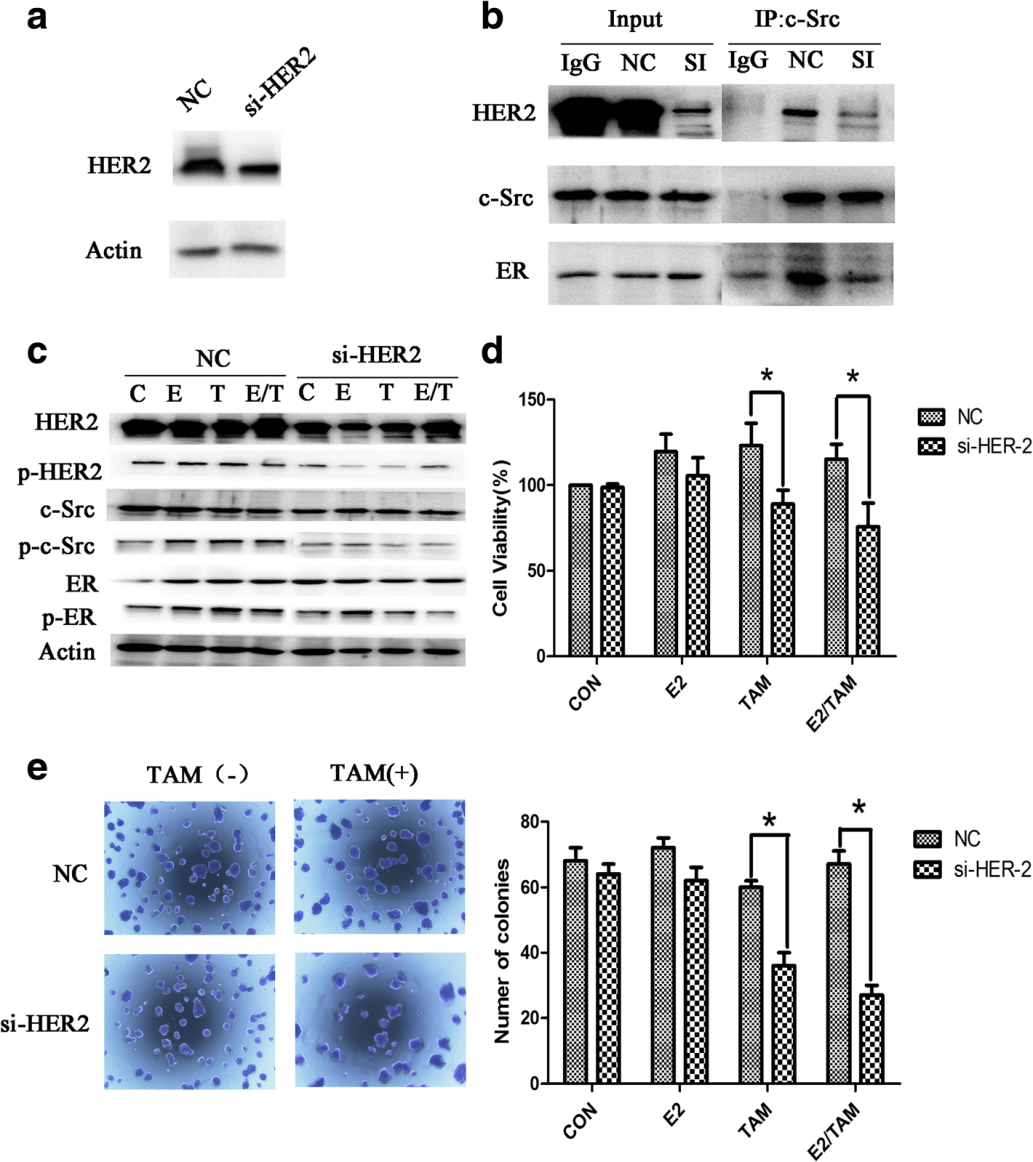
Knockdown of HER2 expression in BT474 cells suppresses the formation of the ER-c-Src-HER2 complex and partially restores tamoxifen sensitivity. **a** Western blot analysis of BT474 cells transfected with siRNA targeting HER2. **b** BT474 cells transfected with HER2 siRNA were subjected to immunoprecipitation and immunoblotting as indicated. **c** Western blot analysis of phosphorylated HER2, c-Src, and ER in BT474 cells knocked down for HER2 expression for 48 h and then treated with 17β-E2 (10 nmol/L) and/or tamoxifen (TAM) (1 μmol/L) for 4 h. **d** MTT assays in BT474 cells transfected with siRNA targeting HER2 for 24 h and then treated with 17β-E2 (10 nmol/L) and/or tamoxifen (TAM) (1 μmol/L) for another 48 h. **e** Colony formation assays of BT474 cells transfected with siRNA targeting HER2 for 24 h and treated with 17β-E2 (10 nmol/L) and/or tamoxifen (TAM) (1 μmol/L) for 14d

To examine the role of HER2 in the formation of the ER-c-Src-HER2 complex, we used siRNA to knockdown the expression of HER2 in BT474 cells. Knockdown of HER2 in BT474 cells (Fig. 4a) inhibited ER-c-Src-HER2 complex formation, the amount of HER2 and ER bound to c-Src was decreased (Fig. 4b). The phosphorylations of HER2, c-Src, and ER were also reduced in HER2 siRNA-transfected BT474 cells (Fig. 4c). We also observed that BT474 cells with HER2 knockdown showed increased sensitivity to tamoxifen compared with controls using cell viability and colony formation assays (Fig. 4d and e). Together these results indicated that suppression of HER2 expression partially reversed the resistance of BT474 cells to tamoxifen. In HER2 si-RNA transfected cells, c-Src-ER complex formation also be inhibited.

**Fig. 5:**
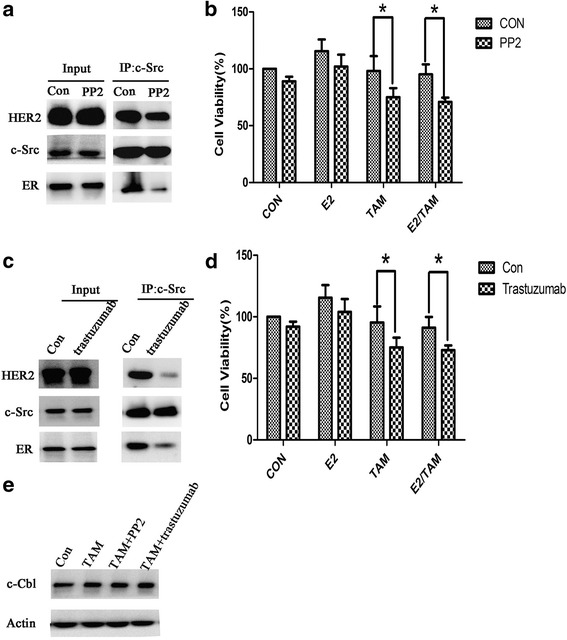
PP2 and trastuzumab inhibit the formation of ER-c-Src-HER2 and partially restore tamoxifen sensitivity. **a** BT474 cells were treated with PP2 (10 μM) for 4 h and subjected to immunoprecipitation and immunoblotting as indicated. **b** MTT assays in BT474 cells treated with PP2 (10 μM) for 4 h and then treated with 17β-E2 (10 nmol/L) and/or tamoxifen (1 μmol/L) for 48 h. *P < 0.05. **c** BT474 cells were treated with trastuzumab (10 μg/ml) for 4 h and subjected to immunoprecipitation and immunoblotting as indicated. **d** MTT assays in BT474 cells treated with trastuzumab (10 μg/ml) for 4 h and then treated with vehicle (CON), 17β-E2 (E2) (10 nmol/L), tamoxifen (TAM) (1 μmol/L) or combination treatment (E2/TAM) for 48 h. *P < 0.05. **e** Western blot analysis of c-Cbl protein in BT474 cells treated with tamoxifen (TAM) (1 μmol/L), TAM (1 μmol/L) combined with PP2 (10 μM), or TAM (1 μmol/L) combined with trastuzumab (10 μg/ml)

We next examined the ER-c-Src-HER2 complex formation in BT474 cells treated with the src inhibitor PP2 or the HER2 antibody trastuzumab and found that complex formation was decreased in response to both treatments (Fig. 5a, c). Furthermore, these agents could partially reverse tamoxifen resistance in BT474 cells (Fig. 5b, d). We also examined the levels of c-Cbl protein in cells treated with PP2 or trastuzumab along with TAM, but we did not observe any obvious difference between control and treatment groups (Fig. 5e).

### Inhibition of lipid rafts reduced ER-c-Src-HER2 complex formation and reversed the resistance of BT474 cells to tamoxifen

**Fig. 6:**
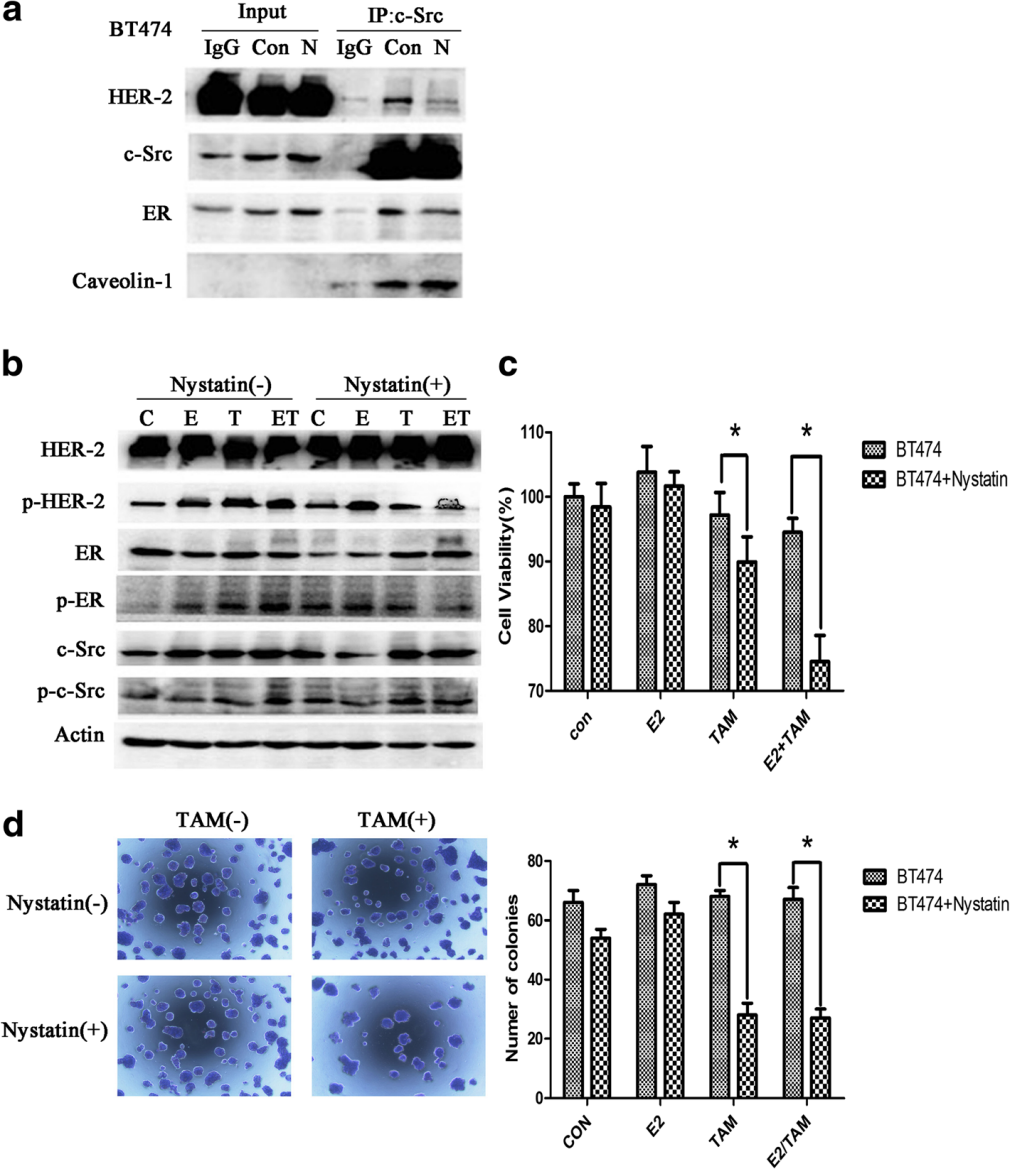
Inhibition of lipid rafts suppresses the formation of the ER-c-Src-HER2 complex and partially restores tamoxifen sensitivity. **a** BT474 cells were treated with vehicle (Con) or nystatin (N) (5 μg/ml) for 2 h and subjected to immunoprecipitation and immunoblotting analysis as indicated. **b** Cells were seeded in 6-well plates (2.5 × 10^4^ cells/well) for 24 h and then treated with vehicle or nystatin(5 μg/ml) for 2 h, followed by treatment with vehicle, E2(10 nmol/L), TAM(1 μmol/L), or the combination for 4 h. **c** Cells were seeded in 96-well plates (5000 cells/well). At 24 h after plating, cells were treated with vehicle or nystatin (5 μg/ml) for 2 h, and then treated with vehicle, E2(10 nmol/L), TAM(1 μmol/L), or the combination. After 48 h, MTT assays were performed. The bar graph shows the sensitivity of BT474 cells to tamoxifen before and after treatment with nystatin. Data represent the mean ± SD of at least three independent experiments. *P* values were determined by the Student’s t test. (*p < 0.05) **d** BT474 cells were treated with nystatin (5 μg/ml) for 2 h, and then treated with E2 (10 nmol/L) and/or TAM (1 μmol/L). Cells were plated in triplicate. On day 14, images were captured and the number of colonies was determined. Data represent the mean ± SD of at least three independent experiments. *P* values were determined by the Student’s t test. (*p < 0.05)

Nystatin inhibits lipid rafts by affecting lipid metabolism. Thus, we next examined the impact of inhibiting lipid rafts by nystatin on ER-c-Src-HER2 complex formation. Treatment of BT474 cells with nystatin reduced the levels of HER2 and ER bound to c-Src (Fig. 6a) and decreased the phosphorylation of HER2 compared with the control group (Fig. 6b). Furthermore, treatment of BT474 cells with nystatin increased the sensitivity of these cells to tamoxifen compared with the untreated cells (Fig. 6c). Colony formation assays showed that BT474 cells with nystatin treatment exhibited fewer colonies than the control group, and the number was further decreased in cells treated with tamoxifen and nystatin combined (Fig. 6d). Together this indicates that nystatin leads to a reversal of tamoxifen resistance in BT474 cells.

### Overexpression of c-Cbl reversed tamoxifen resistance in HER2 overexpressing breast cancer cells

**Fig. 7:**
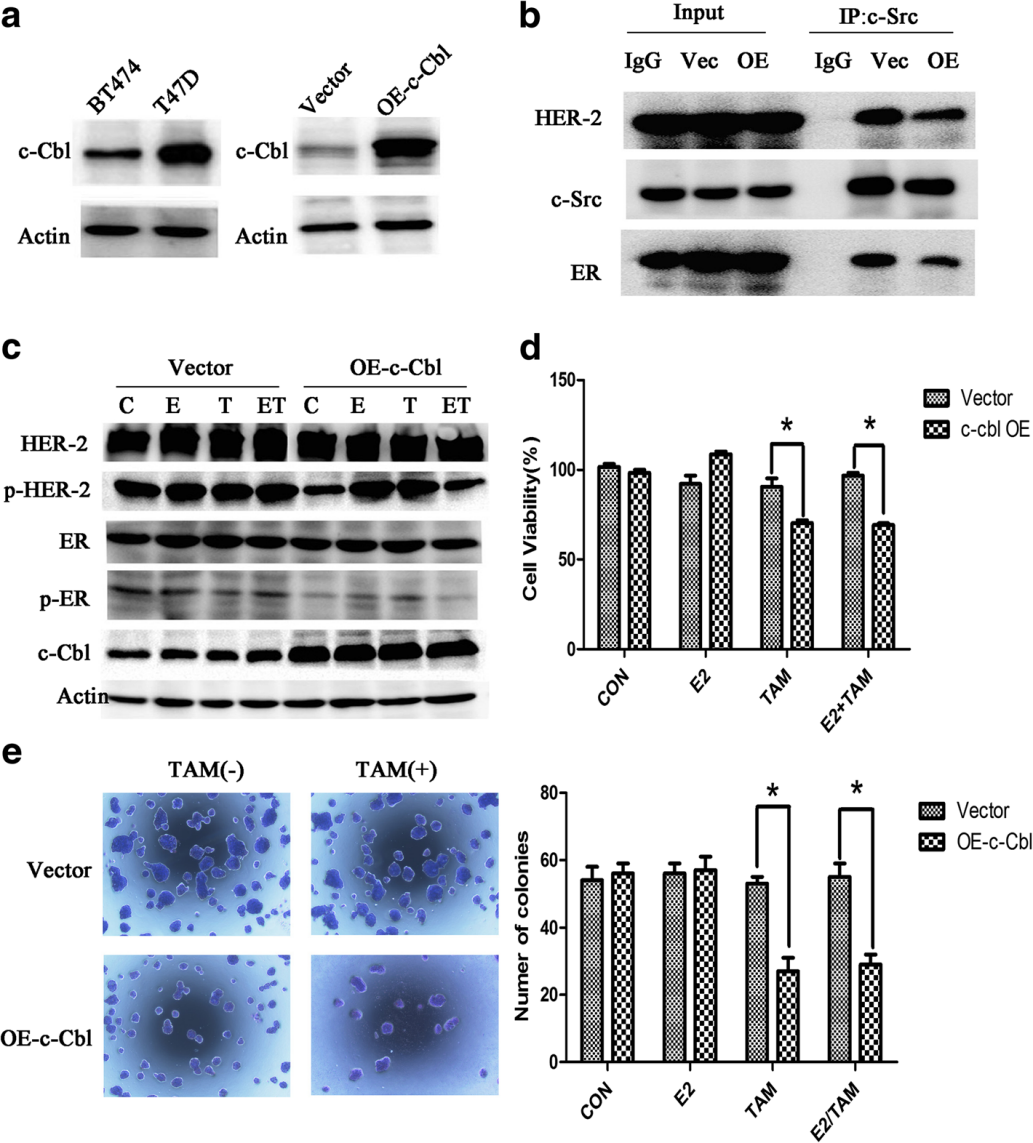
Overexpression of c-Cbl reverses HER2-mediated tamoxifen resistance. **a** c-Cbl protein level was detected in BT474 and T47D cells. And then, BT474 cells were transfected with 3 × flag-CMV-9-c-Cbl (OE-c-Cbl) or 3 × flag-CMV-9 vector(Vector), and then examined the c-Cbl level by immunoblot analysis. **b** Immunoprecipitation after overexpression c-Cbl 48 h in BT474. **c** BT474 cells were transfected with control vector or c-Cbl overexpression plasmids, 24 h later, followed by vehicle, estrogen(10 nmol/L), and tamoxifen (1 μmol/L) or combination treatment for 4 h. Cell lysates were examined by immunoblot analysis using the indicated antibodies. **d** BT474 cells were transfected with plasmids expressing c-Cbl for 24 h, and then exposed to vehicle, estrogen(10 nmol/L), or tamoxifen(1 μmol/L) treatment for another 48 h. Total viable cell number was measured by MTT assays. Data represent the average of three independent replicates ± SD. (*p < 0.05) (**e**) Colony formation assays in BT474 cells transfected with vector or plasmids expressing c-Cbl. BT474 cells were transfected with vector or plasmids expressing c-Cbl for 24 h and then treated with E2 (10 nmol/L) and/or TAM (1 μmol/L) for 14 d. On day 14, colonies were fixed and stained with Giemsa. *P < 0.05

**Fig. 8:**
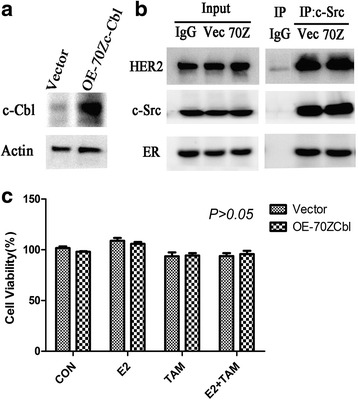
Overexpression of c-Cbl 70Z in BT474 cells. **a** BT474 cells were transfected with PSVL-70Z-c-Cbl (OE-70Zc-Cbl) or PSVL vector (Vector) and c-Cbl level was examined by immunoblot analysis. **b** BT474 cells were transfected with PSVL vector (Vec) or PSVL-70Zc-Cbl (70Z) for 48 h and then examined by immunoprecipitation and immunoblot analysis. **c** BT474 cells were transfected with PSVL-70Zc-Cbl for 24 h and then treated with vehicle, estrogen (10 nmol/L), or tamoxifen (1 μmol/L) for another 48 h. Total viable cell number was measured by MTT assays. Data represent the average of three independent replicates ± SD. *P < 0.05

We found that c-Cbl expression was higher in T47D cells than in BT474 cells (Fig. 7a), indicating a difference in c-Cbl expression levels between tamoxifen resistant and sensitive cell lines. We next examined the potential role of c-Cbl in impacting ER-c-Src-HER2 complex formation by transfecting BT474 cells with a c-Cbl expression vector (Fig. 7a). Overexpression of c-Cbl in BT474 cells reduced ER-c-Src-HER2 complex formation (Fig. 7b) and the phosphorylation of HER2 and ER (Fig. 7c). Furthermore, overexpression of c-Cbl partly reversed the resistance of BT474 cells to tamoxifen (Fig. 7d and e). To determine whether the ubiquitin ligase activity was required for c-Cbl-mediated effects, we used a plasmid expressing the c-Cbl 70Z mutant (Fig. 8a) that lacks ubiquitin ligase activity [23]. Transfection of the c-Cbl 70Z mutant in the BT474 cells did not impact the formation of the ER-c-Src-HER2 complex (Fig. 8b) or tamoxifen resistance (Fig. 8c).

**Fig. 9:**
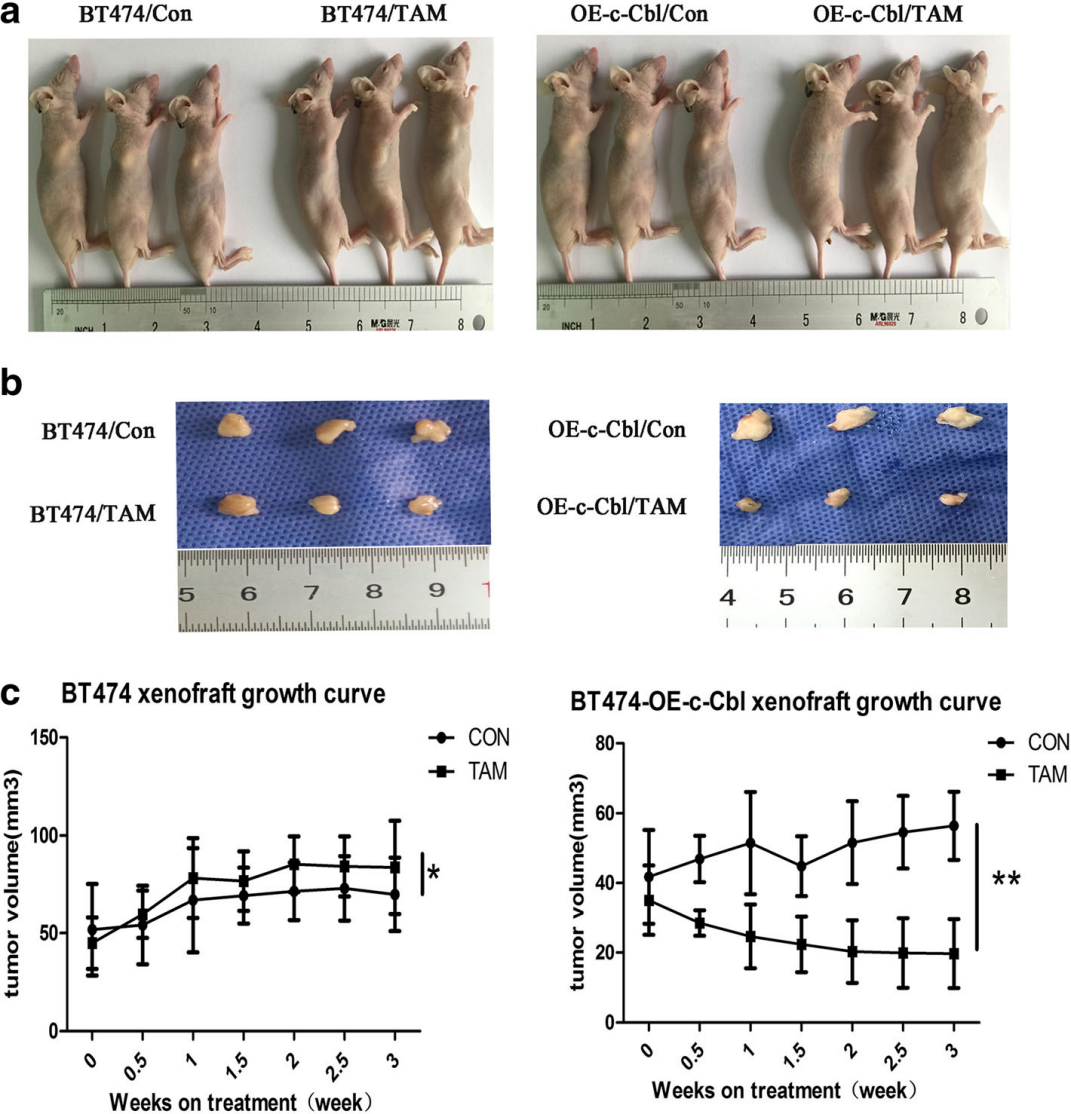
In vivo xenograft nude mouse model. **a** Female athymic mice were injected with BT474 cells or BT474 cells infected with lentivirus expressing c-Cbl (BT474-OE-c-Cbl cells) and then randomized to vehicle or 20 mg/kg TAM groups. Treatment was administered for 7 days. Three mice were included in each treatment group. **b** Xenograft tumors stripped from the nude mice (**c**) Tumor growth curve. Tumors were measured twice per week with calipers. Each data point represents the mean tumor volume in mm^3^ ± SEM. (*P > 0.05,** P < 0.05)

To clarify the role of c-Cbl in tamoxifen resistance, we established a nude mouse xenograft model. We used a lentivirus system to generate BT474 cells that stably overexpressed the c-Cbl protein (OE-c-Cbl cells), and these cells were inoculated subcutaneously into nude mice. BT474 cells were injected into the control group mice. After subcutaneous tumor formation, each group was divided into two subgroups and treated with vehicle or tamoxifen for 7 days (Fig. 9a). Tumor volume was obviously smaller in the c-Cbl overexpression group after tamoxifen treatment compared with control group with tamoxifen (Fig. 9b). The growth curve of transplanted tumors showed that after overexpression c-Cbl, the xenografts were more sensitive to tamoxifen, and the difference was statistically significant (*p* < 0.05, Fig. 9c). These results suggested that overexpression of c-Cbl reversed the resistance of BT474 cells to tamoxifen in vitro and in vivo.

**Fig. 10:**
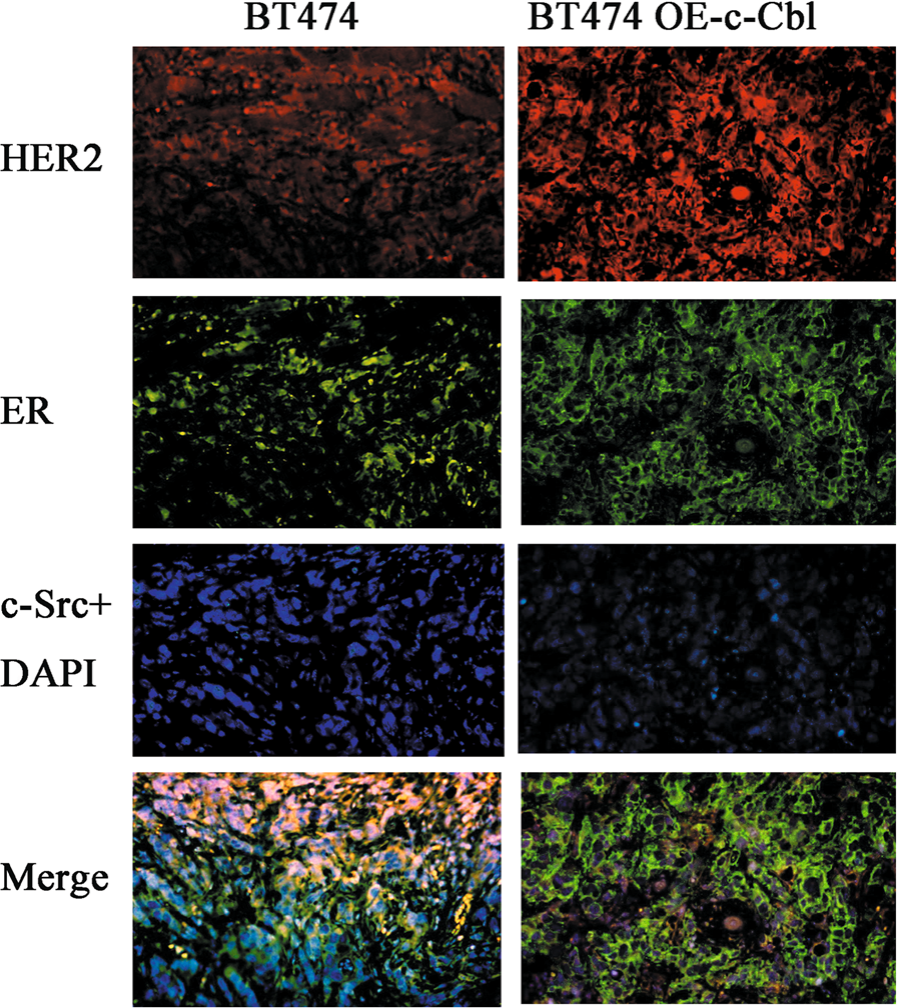
Immunofluorescence of tumor sections from xenograft mice. Formalin-fixed, paraffin-embedded tumors were cut into 4 μm sections and then stained with anti-HER2 (red), anti-c-Src (blue), and anti-ER (green) as indicated. Magnification 200X

We evaluated the expressions of HER2, c-SRC and ER in the mouse tumor samples using immunofluorescence staining. We found that ER, c-Src and HER2 proteins showed co-localization in subcutaneous xenograft tumors formed by wild-type BT474 cells, but the amount of co-localization was reduced in OE-cbl-BT474 subcutaneous xenograft tumors (Fig. 10).

## Discussion

Innate and acquired tamoxifen resistance is an important problem in ER+ breast cancer. HER2 is overexpressed in approximately 15%–30% of human breast cancers and plays a role in tamoxifen resistance [24]. In the previous study, the mechanisms of tamoxifen resistance have been explord, the reports included that pharmacologic mechanisms, loss or modification in estrogen receptor expression, alterations in co-regulatory proteins, autophagy, microRNA, and tumor microenvironment [25–27]. However, the underlying mechanism remains unclear.

BT474 is an ER- positive invasive human breast ductal carcinoma cell line with very high HER2 expression, which is very sensitive to Herceptin but resistant to tamoxifen [28]. We confirmed HER2-overexpressing breast cancer BT474 cells are relatively resistant to tamoxifen. The ER-c-Src-HER2 complex was detected in BT474 cells; this complex participated in tamoxifen resistance, inhibiting the formation of this complex reversed tamoxifen resistance. In Fig. 4, E2 along does not have much effect on cell proliferation after knockdown HER2, but this does not mean that the effect of tamoxifen is independent of ER in BT474 cells. Many previous studies have confirmed the reactivity of BT474 cells to estrogen [29–31]. In our study, the MTT assays were conducted in BT474 cells transfected with siRNA targeting HER2 24 h, and treated with 17β-E2 (10 nmol/L) and/or tamoxifen (TAM) (1 μmol/L) for another 48 h. BT474 is a cell that doubles for a long time. It takes about 96 h to multiply. So in this experiment, the effect of estrogen on the proliferation of BT474 cells is not yet fully reflected.

Lipid rafts are required for HER2 activation and signal transduction [32]. However, whether lipid rafts play a role in the formation of an ER-c-Src-HER2 complex has not been reported. In this study, we showed that lipid rafts are essential for the formation of the ER-c-Src-HER2 complex, as inhibition of lipid raft formation by nystatin reduced ER-c-Src-HER2 complex formation and reversed tamoxifen resistance. Our findings indicated that formation of the ER-c-Src-HER2 complex may play a key role in the crosstalk between ER and HER2 signaling pathways, and our results provide new insight into the mechanism of tamoxifen resistance.

Studies have shown that c-Cbl selectively regulates receptor translocation into lipid rafts [33], and c-Cbl is involved in the regulation of lipid rafts in several cell types [34, 35]. Overexpression of c-Cbl depleted Lck from lipid rafts in Jurkat cells [36]. Our previous study showed that the Cbl ubiquitin ligase family inhibits the function of lipid rafts [9, 16]. In vitro and in vivo experiments confirmed that overexpression of c-Cbl reduced ER-c-Src-HER2 complex formation and reversed tamoxifen resistance in BT474 cells. We also found that the ubiquitin ligase activity of c-Cbl may play a key role in the reversal of tamoxifen resistance. One hand, c-Cbl may reduce the formation of ER-c-Src-HER2 complex through inhibition of lipid rafts aggregation; the other hand, c-Cbl may promote ER-c-Src-HER2 complex ubiquitination through its ubiquitin ligase activity. Therefore, c-Cbl may be used as a therapeutic target in breast cancer patients resistant to tamoxifen, and the regulation of c-Cbl expression should be study furthermore.

## Conclusions

**Fig. 11:**
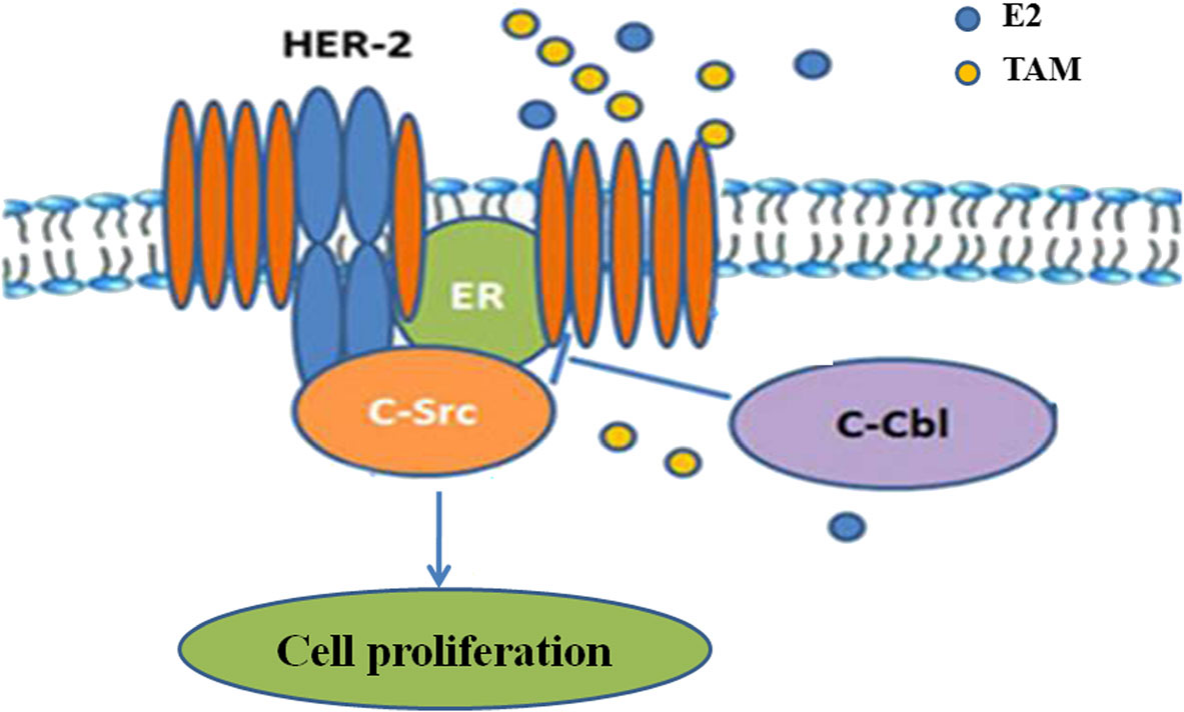
Proposed model for the role of c-Cbl in tamoxifen resistance of HER2-overexpressing breast cancer cells. In HER2-overexpressing breast cancer cells, ER-c-Src-HER2 complex formation results in HER2 signaling pathway activation and tamoxifen resistance. c-Cbl reduces ER-c-Src-HER2 complex formation by inhibiting lipid rafts and restores the sensitivity to tamoxifen

Our results showed that in HER2 overexpressing breast cancer cells, ER-c-Src-HER2 complex formation resulted in HER2 signaling pathway activation and tamoxifen resistance. c-Cbl reduced ER-c-Src-HER2 complex formation by inhibiting lipid rafts, resulting in the reversal of tamoxifen resistance (Fig. 11). Together these findings indicate that ER-c-Src-HER2 complex formation plays a key role in promoting the activation of HER2 and tamoxifen resistance.

## Additional file

Additional file 1: **Figure S1.** Quantification of p-HER2 and p-ER blots in Fig. 7c. BT474 cells were transfected with control vector or c-Cbl overexpression plasmids for 24 h, followed by vehicle (CON), estrogen (10 nmol/L) (E2), tamoxifen (1 μmol/L) (TAM) or combination treatment (E2/TAM) for 4 h. (A) The relative density of phospho-HER2. *P < 0.05. (B) The relative density of phospho-ER. *P < 0.05. (JPG 799 kb)

## Abbreviations

Cbl: Casitas B-lineage lymphoma
E2: 17beta-estradiol
ERα: Estrogen receptor alpha
HER2: Human epidermal growth factor receptor 2
