# Supplementary figures and images for: C-Cbl reverses HER2-mediated tamoxifen resistance in human breast cancer cells
Source: BMC Cancer. 2018 May 2;18:507. doi: 10.1186/s12885-018-4387-5 (PMC5930956; doi:10.1186/s12885-018-4387-5)

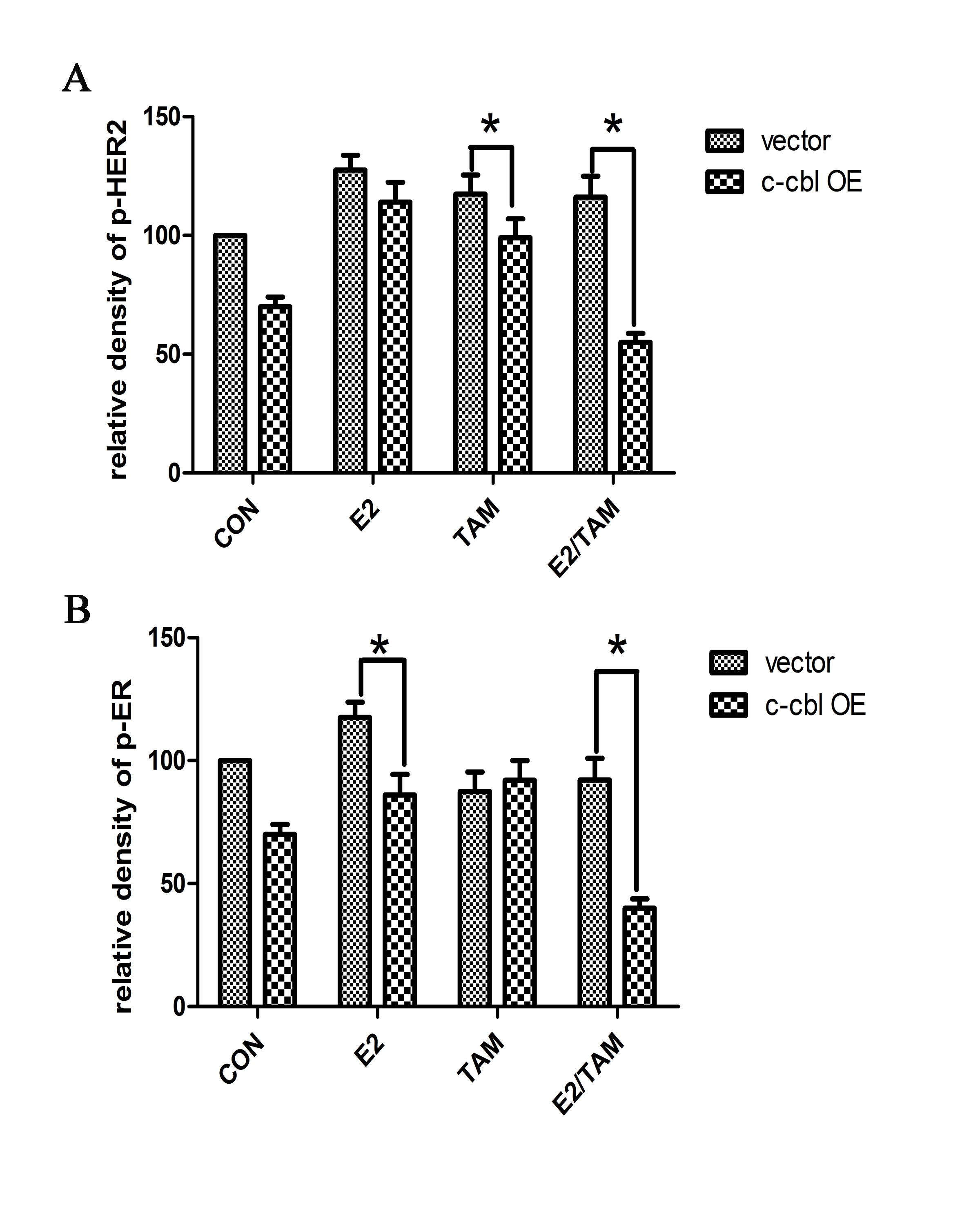

Supplement: Supplementary file 1 — Figure S1. Quantification of p-HER2 and p-ER blots in Fig. 7c. BT474 cells were transfected with control vector or c-Cbl overexpression plasmids for 24 h, followed by vehicle (CON), estrogen (10 nmol/L) (E2), tamoxifen (1 μmol/L) (TAM) or combination treatment (E2/TAM) for 4 h. (A) The relative density of phospho-HER2. *P < 0.05. (B) The relative density of phospho-ER. *P < 0.05. (JPG 799 kb) [file 12885_2018_4387_MOESM1_ESM.jpg]
